# California Residents’ Perceptions of Gene Drive Systems to Control Mosquito-Borne Disease

**DOI:** 10.3389/fbioe.2022.848707

**Published:** 2022-03-10

**Authors:** Cynthia E. Schairer, Cynthia Triplett, Omar S. Akbari, Cinnamon S. Bloss

**Affiliations:** ^1^ Herbert Wertheim School of Public Health and Human Longevity Science, University of California, San Diego, La Jolla, CA, United States; ^2^ Center for Empathy and Technology, Insitute for Empathy and Compassion, University of California, San Diego, La Jolla, CA, United States; ^3^ Section of Cell and Developmental Biology, Division of Biology, University of California, San Diego, La Jolla, CA, United States; ^4^ Department of Psychiatry, University of California, San Diego, La Jolla, CA, United States

**Keywords:** community and stakeholder engagement, public health, vector control, science communication, genetic engineering, gene drives

## Abstract

Scientists developing gene drive mosquitoes for vector control must understand how residents of affected areas regard both the problem of mosquito-borne disease and the potential solutions offered by gene drive. This study represents an experiment in public engagement at an early stage of technology development, intended to inform lab scientists about public attitudes toward their research and inspire consideration and conversation about the social ramifications of creating mosquitoes with gene drive. Online focus groups with California residents explored views on mosquito-borne disease risk, current mosquito control methods, and the proposed development and use of different classes of gene drives to control *Ae. aegypti*. Rather than a dogmatic rejection of genetic engineering or gene drive, many participants expressed pragmatic concerns with cost, control, the ability to narrowly target specific species, and the challenges of mistrust and institutional cooperation. Work like this can inform the alignment of community priorities and the professional priorities of scientists and vector control specialists.

## 1 Introduction

Mosquito vectors of disease represent one of the greatest worldwide threats to human health. Of particular concern is the *Aedes aegypti* (*Ae. aegypti*) mosquito, which can transmit diseases such as Zika, dengue, yellow fever, and chikungunya. This mosquito thrives in urban environments, can live out an entire life cycle indoors, and can lay eggs in very small amounts of water–for example in the tray under a house plant. Because the eggs may dry out and stay viable for over a year, the eggs can hitchhike on objects that once hosted small amounts of dew or rainwater (shipping containers, for example). Due to climate change and global trade, *Ae. aegypti* has appeared in new regions over the past decade, including in California where it was first identified in 2013 (Gloria-Soria et al., 2014; Metzger et al., 2017).

Currently, diseases transmitted by *Ae. aegypti* are rare in California. However, this vector is particularly worrisome to vector control professionals because traditional methods, such as draining standing water, treating large bodies of water with larvicides and mosquito fish, or using repellants and pesticides, are not effective controls. Therefore, there is a need for new approaches to controlling this disease vector. In response, geneticists are developing novel methods for vector control based on new CRISPR-based gene editing techniques, including the use of gene drive to introduce new genetic traits with preferential inheritance into a wild population (National Academies of Sciences Engineering and Medicine, 2016). However, genetically engineered (GE) organisms are controversial, and public support for research on the development of such strategies, particularly in the United States (U.S.), is not well understood.

In 2017 the U.S. Defense Advanced Research Projects Agency (DARPA) created the Safe Genes program, with stated aims of gaining a fundamental understanding of how CRISPR-based gene editing technologies function; devising means to harness them safely, responsibly, and predictably for beneficial ends; and addressing potential health and security concerns related to their accidental or intentional misuse. Team California Safe Gene Drives (hereafter, Team California) was one of the projects funded by this program and aims to safely engineer various classes of gene drive to control the *Ae. aegypti* disease vector. Team California also includes social scientists tasked with investigating the Legal, Ethical, Environmental, Dual-use and Responsible Innovation (LEEDR) dimensions of the technical aims. The technical research is being conducted in public Californian universities and targets a vector present in many parts of the state; therefore, as part of the LEEDR work, we engaged California residents in online focus groups to learn how they responded to the idea of controlling *Ae. aegypti* with gene drive. Here, we report on how these participants discussed the threat of *Ae. aegypti* as well as benefits and concerns associated with proposed GE-based systems with and without gene drive.

This study contributes to the growing literature on public attitudes toward novel forms of vector control and the uses of gene drive. Since the identification of CRISPR systems and their early applications to gene editing, a community of scientists and other stakeholders has rallied to establish paths toward the responsible and safe development of these tools (Oye et al., 2014; Akbari et al., 2015; National Academies of Sciences Engineering and Medicine, 2016; Adelman et al., 2017; Doudna and Sternberg, 2017; Esvelt, 2017; Kuzma et al., 2018; Long et al., 2020). Community and Stakeholder Engagement (CSE) is a cornerstone of these calls for responsible innovation. In addition to facilitating field trials and informing science communication strategies, CSE can help scientists and developers gain and maintain awareness of the needs and desires of those who will likely be affected by their products. Each of these goals may require different approaches to CSE (Schairer et al., 2019). Broad public engagement is only one form of CSE and requires specialized social science methods appropriate for collecting perspectives from a large and diverse set of people. To this end, public engagement often takes the form of surveys and public opinion polls conducted to establish political will and influence policy debates, as well as inform more democratic scientific processes. (Pew Initiative on Food Biotechnology, 2004; Marshall et al., 2010; Ernst et al., 2015; Glenza 2016; Hudson et al., 2019; Jones et al., 2019; MacDonald et al., 2020a; MacDonald et al., 2020b; MacDonald et al., 2021; Schairer et al., 2021).

The LEEDR activities conducted by Team California represent an experiment in public engagement at an early stage of technology development, intended to inform lab scientists about public attitudes toward their research and inspire consideration and conversation about the social ramifications of creating mosquitoes with gene drive. We aimed to collect information from California residents about 1) the perceived acceptability of the gene drive systems being developed by Team California and 2) whether there are specific experiments or design criteria that could be added to the Team California research plan that would address any concerns expressed by Californians. To do so, we held a series of online chat-based focus groups that allowed us to collect responses from a larger and more geographically diverse group of people than would typically attend traditional community meetings or public lectures. The focus group format also allowed us to encourage and center the candid responses of participants in a way that is not possible in other public fora.

Here we present a qualitative analysis of the data collected from these focus groups, comparing the benefits of and concerns about GE and gene drive mosquitoes discussed by participants.

## 2 Materials and Methods

### 2.1 Participants

This project was designed as a program evaluation of the experimental gene drive systems being developed at the University of California, with the goal to provide scientists with feedback from the public. The protocol was reviewed and approved by the institutional review board at the University of California, San Diego (project #170944). In program evaluation, data collected are about the program rather than about the participants and therefore obtaining formal consent is not considered necessary.

To reach a cross-section of Californians, we contracted with Ipsos (formerly GfK Custom Research) to recruit focus group participants from their national probability-based online panel (GFK Knowledge Panel). We asked Ipsos to recruit English-speaking participants based on education level (with or without a Bachelor’s degree) and proximity to counties in which *Ae. aegypti* are known to be present. English-speakers from zip codes with a population density over 45 people/square mile were invited to focus groups according to their level of education and the presence or absence of *Ae. aegypti* in their county. Presence of *Ae. aegypti* was determined based on reports from the California Department of Public Health (California Department of Public Health, 2019). We planned three focus groups for each cohort but added groups to supplement for low enrollment. Due to the logistical constraints in recruitment and scheduling, Spanish-speakers were not divided according to education and location but instead invited from all California zip codes. Overall, we conducted a total of 18 focus groups (Table 2).

Dividing the focus group participants according to education or language preference was intended to create some degree of affinity among participants per best practices in focus group design (Barbour, 2007). We clustered participants based on presence or absence of *Ae. aegypti* to be sure that we heard from people who might be directly affected by novel vector control technologies and those who would be more indirectly affected. Because of the large Spanish-speaking population in California, we felt it was especially important to include this group. As this was conducted as a program evaluation, we did not collect individual-level demographics.

### 2.2 Focus Group Format

**FIGURE 1:**
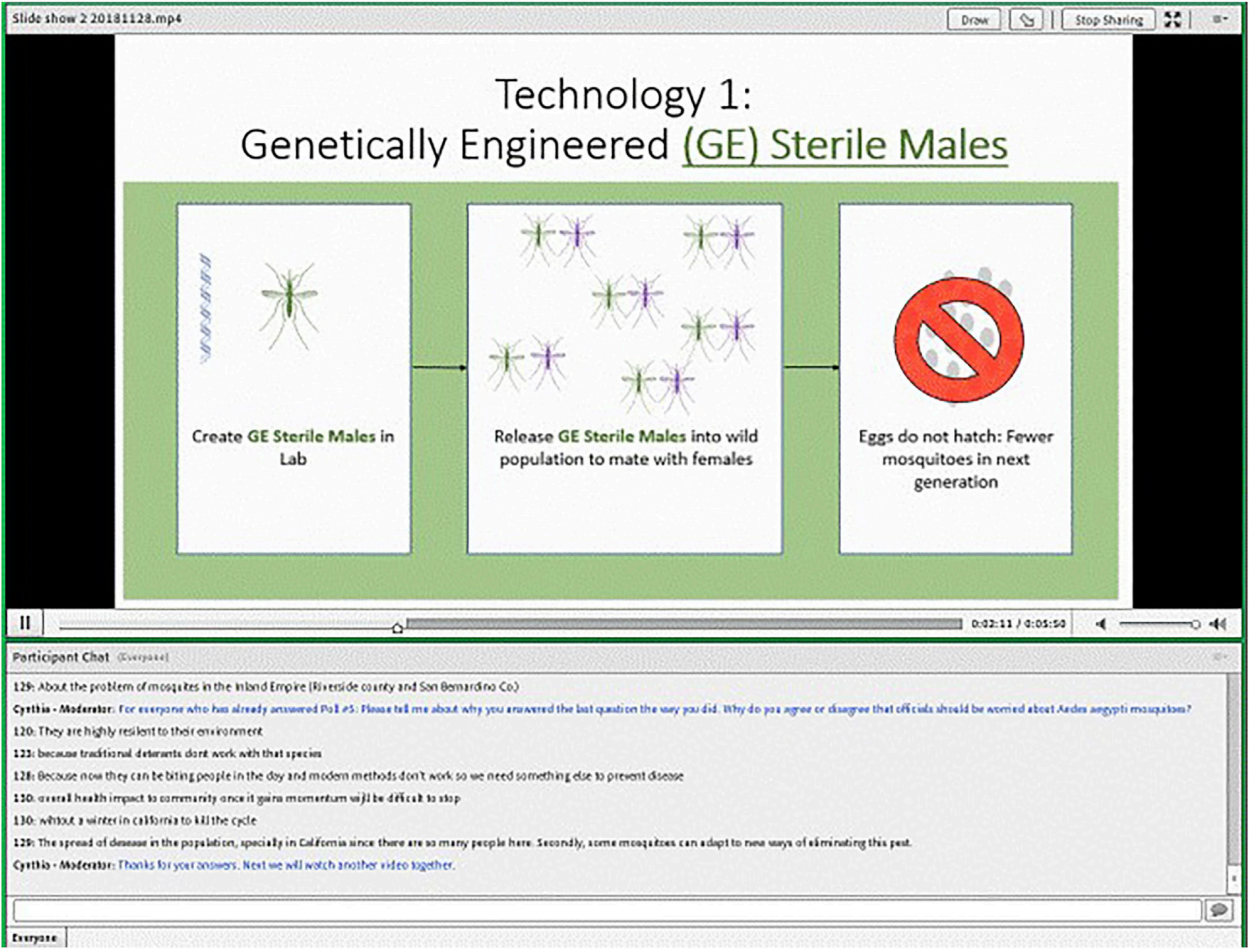
Screenshot of FocusVision interface, participant view.

We elected to conduct our online focus groups using text-chat instead of video to maintain a high level of privacy for respondents. We used the online platform, FocusVision, made available through a partnership with Ipsos. The interface allowed us to simultaneously present videos or images, ask fixed-choice polling questions, and facilitate a group chat (see Figure 1).

Traditionally, in-person focus groups have been convened to record talk and interactions among a group of people over a topic already familiar to them (Barbour, 2007; Macnaghten, 2017). However, we sought to use online focus groups as an “anticipatory method” (Macnaghten, 2017) to investigate public responses to novel emerging technologies. While GE for vector control has received some media attention, reports have not been frequent enough nor of sufficient general interest to be considered common knowledge. Therefore, a primary challenge in collecting public responses to these techniques was presenting accessible and reasonably unbiased information about a rapidly emerging technology in a new field where there is still disagreement among experts (Yeo and Brossard, 2017; Brossard et al., 2019).

We devoted considerable time to creating informational narrated slideshows through a close collaboration between members of the Bloss and Akbari labs, that is described in detail elsewhere (Schairer et al., 2020). The focus group protocol was organized around four narrated slideshow videos covering 1) mosquitoes in California and basic mosquito facts; 2) a comparison of the GE-based sterile insect technique (GE-SIT) and GE mosquitoes with gene drive; 3) a comparison of gene drive mosquitoes designed to suppress populations versus gene drive mosquitoes designed to modify populations; and 4) a comparison of different types of control strategies for gene drive mosquitoes (self-limiting, threshold-dependent, and self-sustaining with callback measure). The topics, total number of slides, duration of each video, and the number of forced choice polling questions included in each section are presented in Table 1.

**TABLE 1:** Structure of chat-based focus group sessions.

Sequence	Title/Theme	Slideshow duration (minutes)	Number of slides	Forced choice polling questions	Open discussion prompts
Opening	Initial Perceptions of the Problem	—	1	2	3
Slide Show 1	“Mosquitoes in California”	5:10	10	3	2
Slide Show 2	“Genetic Engineering for Mosquito Control”	5:50	8	4	2
Slide Show 3	“Modifying Mosquitoes with Gene Drive”	2:49	5	2	1
Slide Show 4	“Controlling Gene Drives”	5:49	8	4	2
Closing	Review and Discussion	—	—	4	3

The GE sterile male system discussed in the slideshow was based on precision guided sterile insect technique (pgSIT) proposed by Kandul and colleagues (Kandul et al., 2019). Similar to traditional sterile insect technique (SIT) where radiation is used to produce sterile insects, pgSIT introduces sterile males to the environment to mate with wild mosquitoes resulting in non-viable eggs and reducing the overall population. Unlike traditional SIT, however, pgSIT uses GE to produce mosquito eggs that, when hydrated, will only hatch sterile male and intersex mosquitoes. The gene drive systems presented in the slideshows were based on proposals to introduce lethal genes that could theoretically eliminate an entire wild population over time (Kyrou et al., 2018). The slideshows discussed “local authorities” as the potential future users of these technologies.

Between each video, the moderator presented participants with a combination of polling questions and open-ended questions in a chat box. The polling questions had fixed response options and were designed to start conversations and contextualize the open-ended answers. Slideshow videos and a listing of the polling and discussion questions are posted and published (Bloss, 2018a; Bloss, 2018b; Bloss, 2018c; Bloss, 2018d; Cheung et al., 2020; Schairer et al., 2020).

### 2.3 Analysis

The videos and polling questions created a standard structure across all 18 focus groups that we exploited in the analysis as a way to systematically break up the transcripts and compare responses across all focus groups. We categorized text chat answers following Slideshow 1 according to a set of common themes about the threat of mosquitoes, and the text chat following Slideshows 2 through 4 according to a set of common themes about mosquito control strategies using GE and gene drive. Themes included types of information that participants noted as interesting or surprising (e.g., that male mosquitoes do not bite); noted features of GE mosquitoes (e.g., their ability to target one species); and common concerns (e.g., impact on local ecosystems). For ease of reading, we have edited quotes for spelling, punctuation, and capitalization, including accents and special characters for Spanish quotes. Any grammar changes or additional words added for clarity appear in brackets.

## 3 Results

One-hundred-thirty-six (136) individuals participated in 18 focus groups. All recruited participants lived in different zip codes. Table 2 presents the number of focus groups held for each cohort and the number of participants in each group. In response to polls before any information was shared, 45.6% of respondents thought mosquitoes were a problem in their area and 50.7% thought they were not a problem (3.7% did not answer this poll); 53.7% of respondents were aware of a vector control agency in their area and 38.2% were not. We did not observe any consistent qualitative differences between the cohorts and there were no statistically significant differences between groups living or not living in areas affected by *Ae. aegypti* with respect to any of the polling data.

**TABLE 2:** Number of focus groups and participants in recruitment cohorts.

	*Ae. aegypti* not reported		*Ae. aegypti* reported		Total	
	Focus groups	Participants	Focus groups	Participants	Focus groups	Participants
Less than a Bachelor’s degree	3	29	4	25	7	54
More than a Bachelor’s degree	3	33	3	20	6	53
Spanish Speakers	—	—	—	—	5	29
Total	6	62	7	45	18	136

### 3.1 Considerations of GE and Gene Drive Mosquitoes

After both GE and gene drive methods to control mosquitoes were presented to these groups, the focus of comments moved freely between genetic engineering generally (including gene drive as a subset of GE) and direct comparisons of GE-SIT with gene drive mosquitoes. Participants noted appealing features and concerns that apply to all GE systems and some that apply differently to GE-SIT and gene drive systems. Table 3 summarizes these features and concerns.

**TABLE 3:** Comparison of appealing features and concerns raised by focus group participants for the presented technologies.

System	Appealing features	Concerns
Any GE	•Not pesticides	•Will not be ready before disease arrives
	•Targeted	•Skepticism and desire for more information
	•Does not require individual action (like vaccines)	•Unwanted environmental outcomes
		•Effects on human health (being bitten or pathogen mutation)
		•Barriers to public acceptance
		•Distrust of government and industry
		•General discomfort with GE
GE-SIT	•Control and confinement clear and intuitive	•Expense (many releases)
	•Local decision to use	
Gene Drive	•Cost effective (fewer releases)	•Requires geo-political cooperation
		•May require large release

#### 3.1.1 Appealing Features

Participants pointed to two features of GE systems as particularly appealing: that they work without the use of pesticides and that, unlike pesticides, they target only specific species. For example, after viewing Slideshow 2, one participant commented, “Finally! A solution that doesn’t require spraying dangerous pesticides all over the city. Can’t wait for them to do this” (204BA-/Aa+). Another said, “*Sería mejor que lo que hicieron en los 80s que traían avionetas fumigando y dañando nuestra salud*” ([Gene drive] would be better than what they did in the 80s when they brought in planes fumigating and damaging our health.) (912S). The preference over pesticides continued to be expressed after Slideshows 3 and 4, culminating in the answers to a poll at the end of the sessions where 125 respondents (92%) indicated that genetic engineering would be “better” than pesticides while only seven indicated that it would be “worse.” We also saw many discussions about the ability of genetic engineering approaches to target single species. Some participants wanted to clarify that this would be the case, often asking questions about breeding behavior among mosquitoes. For example, one participant asked, “can they leave all other kinds alone?” (775BA-/Aa-) and another asked, “¿*Como reaccionarán los mosquitos hembras con estos mosquitos modificados? ¿Se aparearán de la misma manera?*” (How do female mosquitoes react to the modified mosquitoes? Will they still mate the same way?) (738S).

As participants made sense of the differences between the GE-SIT system and the various gene drive systems presented throughout the session, many participants were particularly interested in the relative cost of the methods. For example, one participant commented, “Both seem like viable solutions. My deciding factor would be the price point” (559BA+/Aa-). Some focused on the fact that, because gene drive could potentially work after only one release, this method would be more cost effective, asking versions of the question, “Is it [GE-SIT] economically feasible?” (788BA-/Aa-).

Control was also top of mind for many when comparing the methods that were presented. For example, a participant commented, “I’d say I’m more ok with GE sterile [GE-SIT] because there’s more opportunities to stop it if something bad were to happen with the gene editing” (201BA-/Aa+). A similar concern was geographical control or confinement of gene drive systems that might, in theory, lead to the eradication of a population after only one release. Regarding gene drive, one participant asked, “¿*Cómo se controla la populación en un área? Estos vuelan de zona a zona, estado a estado,*” (How do you control the population in an area? These [mosquitoes] fly from area to area, state to state) (2036S). With respect to control and confinement, the GE-SIT system had the attractive feature of a clear way to stop.

When considering the different control strategies for gene drive systems presented in Slideshow 4, the importance of confinement was again discussed. While some were in favor of more controlled methods, 43% of participants selected self-sustaining gene drive (the least controlled option) as the “most acceptable to use” in their communities in response to a poll. At the same time there were some discussions of how the use of gene drive might be coordinated across city, county, state, or international borders. In two groups, such responses were accompanied by comments that the decision to use a self-sustaining system would require federal action because individual states or counties would not be able to make the decision on their own. These comments imply that, in contrast, controlled methods could be deployed by local authorities.

#### 3.1.2 Common Concerns and Questions

Most of the concerns raised by participants were applicable to both GE and gene drive systems. While the potential efficacy, cost-efficiency, and control of these systems were appealing, participants also voiced concerns and asked critical questions about whether and how these features will be achieved. These technical concerns included whether the GE systems would be effective in reducing the mosquito population; if the systems would be prohibitively expensive to use; or if they will be developed in time to address vector-borne disease before an outbreak. Some such comments called for more information or research. For example, one participant wondered about how many mosquitoes would be necessary: “My suspicion about gene drive is that research would be required to determine the mating rate and reproductive rate to determine if a huge cloud of GE males would need to be released in order to be effective.” (734BA-/Aa-) Another participant expressed a desire to see “proof that these methods are making a difference without much altering [of] other problems.” (784BA-/Aa-) Other participants wanted more information about the state of the science: *“¿y cuál fue el récord? Dos años es muy corto el plazo para ver verdaderamente las consecuencias.”* (How has this been working out so far? Two years is a very short time to truly see the consequences) (2056S). In these discussions, participants often expressed conditional approval, for example, “If the data provides that it is safe within margins and is double checked by other agencies then fine use it.” (109BA+/Aa+).

Many comments about unwanted outcomes revolved around possible adverse effects on the local ecosystem should *Ae. aegypti* be successfully eliminated. Though the slideshows presented *Ae. aegypti* as non-native to California, participants wondered, “Does AA [*sic*] have any function in our ecosystem or can we get along without it?” (529BA+/Aa-), “Would this have a negative effect on other insects?” (739BA-/Aa-), and “I assume the bugs that eat mosquitoes are just as willing to eat sterile/gene modified ones as not?” (110BA+/Aa+). Some worried about possible dangers related to being bitten by modified mosquitoes and the possibility of either the mosquitoes or the pathogen developing resistance to a genetic intervention. For example, “I am more concerned about what’s in the bite than the bite [itself]” (785BA-/Aa-) and, “What if the diseases evolved to become better at infecting the mosquitos?” (522BA+/Aa-).

Some participants raised concerns about the barriers to public acceptance of these technologies. Participants across groups addressed the importance and cost of public education for both acceptance and cooperation. For example, “Before funding the research [unspecified subject] should let everyone know. And educate them” (760BA-/Aa-). Some worried that the public will not accept these technologies without enough education or that they could undermine the intervention by, for example, killing the GE mosquitoes. As one participant put it, *“Una duda que tengo es si se alertaría a la población para no rociar insecticidas sobre los mosquitos machos.”* (Will the public be instructed not to spray pesticides to combat the male mosquitoes?) (804S).

Other social concerns revolved around who will decide what research to fund or when to use GE mosquitoes. Such discussions often included expressions of mistrust of the government or for-profit companies. For example, “As long as no company can somehow claim any copyright [of] this method or the like.” (109BA + Aa+) One participant voiced questions about the transparency of the focus group itself: *“No es debata, es información, para acudir opinión del público para entonces utilizar como sea adecuada. ¿Quién pagó a [moderator] para ser este proceso?”* (This is not a debate, it is information, in order to get public opinion which will then be used however they see fit. Who paid [moderator] to do this?) (2036S). Another participant felt that educated citizens should be consulted: “Citizen oversight can be a good thing, but the citizens should understand a little about science and the scientific method, and not be employed by the companies providing chemical or modified mosquitoes” (522BA+/Aa-). Another raised questions about how voters may respond to these methods: “I keep asking how many years and voters will complain about cost. Some in big cities may not have the problem but they do vote!” (539BA+/Aa-).

Finally, some participants voiced general discomfort with using GE, and a few expressed outright rejection of GE or questioned the assumption that GE mosquitoes would be better than pesticide use. For example, “Why are we replacing spraying them? Is that worse than changing their DNA?” (539BA+/Aa-). Many others worried about unforeseen consequences connected to gene drive in particular and the possibility of a slippery slope toward other types of gene drive organisms. For example, participants commented, “I don’t have a problem with it if it was only used to control mosquitoes. I have a problem if it starts with mosquitoes and leads to other things” (619BA-/Aa+), and “I am not sure of the risks associated with Gene Driving. It might be a solution or it might also create another problem” (724BA-/Aa-). Some of the participants who voiced these concerns gravitated toward the GE-SIT method. For example, after viewing Slideshow 3, comparing gene drive for population reduction vs gene drive for modification, one participant stated, “Interesting concepts but I always wonder about any unanticipated side effects; I like the GE model” (134BA+/Aa+).

Some focus groups clearly weighed or debated these concerns and questions with reference to the threat of human disease and alternative solutions, such as pesticides or vaccines. As two participants put it, “the fact [that GE mosquitoes are] not hazardous to humans is a plus but not doing anything is the hazard” (703BA-/Aa-), and “Well, hypothetically, any gene modification could have unintended consequences. That doesn’t change the fact there is a threat that needs to be addressed.” (501BA+/Aa-) Another group had an extended exchange about the possibility of pursuing a vaccine for these diseases. One participant contributed, “Well a vaccination sounds good but why put that on humans = there is already the immunization vaccines having issues with parents vs. doctors vs. schools, if we can eliminate humans getting the disease another way I think that is the better option” (703BA-/Aa-).

## 4 Discussion

In these focus groups, California residents engaged in a nuanced consideration of GE and gene drive for mosquito control. Along with positive comments and willingness to consider these technologies came many questions and clarifications that would be critical to address had we been asking participants to make a commitment to any of these methods. Just as participants saw reasons for optimism, they also raised many reasons for caution. The same participants who expressed openness to GE for vector control also often voiced worry and discomfort with the unknowns, possible adverse outcomes, and complexity associated with these methods. When participants discussed possible adverse consequences, they weighed them with their perception of the disease threat and the risks of alternative possible solutions. These comments reflect how California residents consider a broad set of priorities that may differ from the more focused professional priorities of scientists and vector control specialists.

The merits and concerns raised by these California residents are quite similar to those found in other survey, interview, and focus group studies. Other studies of communities or publics consistently discuss hopes that GE and gene drive will provide effective, safe, and economical solutions to the threat of vector-borne diseases (Marshall et al., 2010; Hudson et al., 2019; Jones et al., 2019; Hartley et al., 2021; MacDonald et al., 2021). Likewise, these studies have recorded common concerns related to environmental impact, off-target impacts, human health risks, and general wariness of GE technologies. Concerns about governance (Hudson et al., 2019; Jones et al., 2019; Hartley et al., 2021) and socio-political impact (Marshall et al., 2010; Hudson et al., 2019; Hartley et al., 2021) have featured in only some of these prior studies. We note that cost appeared to be more central to our participants’ considerations than is suggested by the documentation of other studies.

These findings suggest that gene drive systems for mosquito control could find support in California, especially if experts are able to adequately address concerns about the impact of eliminating the target species, cost, and efficacy. Additionally, support of gene drive for mosquito control will likely hinge on awareness of the threat of mosquito-borne disease in California and how addressing this threat ranks among competing priorities. These findings suggest that establishing the threat of these diseases may be enough to engender openness to, if not support for, gene drive systems.

Given the controversy surrounding Oxitec’s trials of GE mosquitoes in Florida (Bloss et al., 2017; Schairer et al., 2021), it is reasonable to wonder if Californians would support the use of gene drive mosquitoes without the presence of endemic mosquito-borne disease. We note that in this study, Slideshow 1 appears to have conveyed to this threat in the course of a 5 min narrated slideshow. It is not clear, however, that such a presentation would effectively convey this in other venues, such as mass media campaigns or community forums. Community leaders and public health officials should not assume that citizens are aware of the threat of mosquito-borne disease in California, nor should they assume citizens will dismiss this threat in the absence of endemic cases.

### 4.1 Limitations

While our sample size would be small for a survey study, our 18 focus groups make this a large qualitative study that captured the geographic diversity within California through online focus groups. As a qualitative study, it was designed to study the presence rather than the prevalence of the opinions and themes we observed. The findings from this study could inform the collection of more generalizable data through a survey of a larger sample.

An important consideration for this study was the content of the narrated slideshows used as stimulus materials for these focus groups. Because the slideshows were prerecorded, they allowed for a uniformity in both content and structure across the groups. However, this also made it more difficult for participants to ask clarifying questions and gave the moderator less flexibility in leading the conversation. Though we worked hard to produce accurate and reasonably neutral content, we acknowledge the possibility of bias in such materials. Importantly, we have made our slideshows publicly available (Bloss, 2018a; Bloss, 2018b; Bloss, 2018c; Bloss, 2018d) and extensively documented the process of development (Schairer et al., 2020).

The chat-based format for these focus groups also created challenges for moderating. Though this format allowed for complete anonymity of the participants, it precluded our ability to use non-verbal cues when interacting with the group. The text-chat allowed for everyone to type at once, which helped us collect many opinions but likely hindered discussion between participants. Some groups seemed to address most of their comments to the moderator, while others did generate conversations and some debate, especially in the second half of the sessions. Some guides on conducting online focus groups suggest asking participants to take turns and mind the interface’s signal that another is typing (Lobe, 2017). Such a practice may have fostered even more discussion, but also would have afforded less time for everyone to contribute.

### 4.2 Implications and Future Directions

Efforts to create more democratic science, inclusive public debate, and community and stakeholder engagement surrounding the use of gene drive rely on diverse groups of people listening to each other and an openness to learn from the experiences of others. This focus group project was one approach to collecting reflections of California residents to share with the scientists who work in public state universities. The hope is that projects like this one will help to build a bridge between scientists and members of the lay public who might not otherwise be able to hear one another. Rather than create a public meeting that centers expert presentations, this focus group approach provided space for participants to ask questions and discuss the presented technology without the potential inhibitions some feel in the presence of experts or in large groups. This approach also allowed us to reach a more geographically diverse group of people.

The findings can inform the work of scientists and gene drive developers by providing insight into how uninitiated California residents respond to the problem of *Ae. aegypti* control and potential solutions being explored in current research. These findings are a reminder that most Californians are unaware of the special challenges related to controlling *Ae. aegypti* that motivate the research of Team California. The study also illustrates the many competing priorities California residents will consider when faced with GE and gene drive solutions. In this case, most participants agreed that the problem was worrisome, despite little prior knowledge, and many were receptive to novel approaches to vector control. However, understanding the problem did not lead to unquestioned acceptance of the proposed possible solutions; participants sought to balance priorities and risks and desired more information and assurances of transparency before supporting any given solution. In addition, participants expressed more concerns about GE mosquitoes in general rather than gene drive specifically. This suggests that resistance to GE technologies as a general category may be more of a barrier than resistance specific to gene drive.

We note that findings from this work have inspired the outline of a set of core commitments for field trials of gene drive organisms (Long et al., 2020). Prior to any gene drive field release, these commitments include fair partnership and transparency, testing of product efficacy and safety, regulatory evaluation and risk/benefit analysis, and developing monitoring and mitigation strategies. Additionally, given the concerns related to non-confinable gene drive technologies observed in this study, the Akbari lab is prioritizing research on confineable technologies for controlling populations such as self-limiting drives (Li et al., 2020) and GE-SIT systems (Li et al., 2021). This is an example of how this work on community and stakeholder engagement has directly informed research and development within Team California.

This study underscores the crucial and on-going work of identifying and aligning the priorities of citizens and professionals in public health efforts. As the research on gene drive mosquitoes progresses, developers and their partners in public health agencies must remember to state the problem they are trying to address and listen to how community members and other stakeholders may weigh the problem in the context of broader considerations. Maintaining awareness of this will help developers to create solutions that are both acceptable and usable for the communities they wish to serve.

## Data Availability

The raw data supporting the conclusion of this article will be made available by the authors, without undue reservation.

## References

[B1] Adelman Z. Akbari O. Bauer J. Bier E. Bloss C. Carter S. R. (2017). Rules of the Road for Insect Gene Drive Research and Testing. Nat. Biotechnol. 35 (8), 716–718. 10.1038/nbt.3926 28787415 PMC5831321

[B2] Akbari O. S. Bellen H. J. Bier E. Bullock S. L. Burt A. Church G. M. (2015). Safeguarding Gene Drive Experiments in the Laboratory. Science 349 (6251), 927–929. 10.1126/science.aac7932 26229113 PMC4692367

[B3] Barbour R. S. (2007). Doing Focus Groups. London: Sage.

[B4] Bloss C. (2018a). Slide Show 1: Mosquitoes in California. Available at: https://tinyurl.com/blossslides1 (Accessed August 6, 2019).

[B5] Bloss C. (2018b). Slide Show 2: Genetically Engineering for Mosquito Control. Available at: https://tinyurl.com/blossslides2 (Accessed August 6, 2019).

[B6] Bloss C. (2018c). Slide Show 3: Modifying Mosquitoes with Gene Drive. Available at: https://tinyurl.com/blossslides3 (Accessed August 6, 2019).

[B7] Bloss C. (2018d). Slide Show 4: Controlling Gene Drives. Available at: https://tinyurl.com/blossslides4 (Accessed August 6, 2019).

[B8] Bloss C. S. Stoler J. Brouwer K. C. Bietz M. Cheung C. (2017). Public Response to a Proposed Field Trial of Genetically Engineered Mosquitoes in the United States. Jama 318 (7), 662–664. 10.1001/jama.2017.9285 28810013 PMC5817561

[B9] Brossard D. Belluck P. Gould F. Wirz C. D. (2019). Promises and Perils of Gene Drives: Navigating the Communication of Complex, post-normal Science. Proc. Natl. Acad. Sci. U S A 116 (16), 7692–7697. 10.1073/pnas.1805874115 30642954 PMC6475393

[B10] California Department of Public Health (2019). *Aedes aegypti* and *Aedes albopictus* Mosquitoes in California. Available at: https://www.cdph.ca.gov/Programs/CID/DCDC/CDPH%20Document%20Library/AedesDistributionMap.pdf (Accessed July 3, 2019).

[B11] Cheung C. Gamez S. Carballar-Lejarazú R. Ferman V. Vásquez V. N. Terradas G. (2020). Translating Gene Drive Science to Promote Linguistic Diversity in Community and Stakeholder Engagement. Glob. Public Health 15, 1551–1565. 10.1080/17441692.2020.1779328 32589115

[B12] Doudna J. A. Sternberg S. H. (2017). A Crack in Creation: Gene Editing and the Unthinkable Power to Control Evolution. Boston: Houghton Mifflin Harcourt.

[B13] Ernst K. C. Haenchen S. Dickinson K. Doyle M. S. Walker K. Monaghan A. J. (2015). Awareness and Support of Release of Genetically Modified "sterile" Mosquitoes, Key West, Florida, USA. Emerg. Infect. Dis. 21 (2), 320–324. 10.3201/eid2102.141035 25625795 PMC4313646

[B14] Esvelt K. (2017). “Rules for Sculpting Ecosystems: Gene Drives and Responsive Science,” in Gene Editing, Law, and the Environment: Life beyond the Human. Editor Braverman I. (London: Routledge), 21–37.

[B15] GFK KnowledgePanel (2019). Be Sure with KnowledgePanel. Available at: https://www.gfk.com/fileadmin/user_upload/dyna_content/US/documents/GfK_Fact_Sheet_-_Knowledge_Panel_-_Overview.pdf (Accessed May 8, 2019).

[B16] Glenza J. (2016). Genetically Modified Mosquitoes Could Be Released in Florida Keys by spring; in Fight against Zika, British Company Oxitec Must Seek Approval from FDA for Insects' Release into the Wild Following Monroe County Referendum. Monroe County, Florida: The Guardian. Available at: https://www.theguardian.com/us-news/2016/nov/26/zika-virus-genetically-modified-mosquitoes-florida .

[B17] Gloria-Soria A. Brown J. E. Kramer V. Hardstone Yoshimizu M. Powell J. R. (2014). Origin of the Dengue Fever Mosquito, *Aedes aegypti*, in California. Plos Negl. Trop. Dis. 8 (7), e3029. 10.1371/journal.pntd.0003029 25077804 PMC4117443

[B18] Hartley S. Smith R. D. J. Kokotovich A. Opesen C. Habtewold T. Ledingham K. (2021). Ugandan Stakeholder Hopes and Concerns about Gene Drive Mosquitoes for Malaria Control: New Directions for Gene Drive Risk Governance. Malar. J. 20 (1), 149. 10.1186/s12936-021-03682-6 33726763 PMC7968178

[B19] Hudson M. Mead A. T. P. Chagné D. Roskruge N. Morrison S. Wilcox P. L. (2019). Indigenous Perspectives and Gene Editing in Aotearoa New Zealand. Front. Bioeng. Biotechnol. 7, 70. 10.3389/fbioe.2019.00070 31032252 PMC6470265

[B20] Jones M. S. Delborne J. A. Elsensohn J. Mitchell P. D. Brown Z. S. (2019). Does the U.S. Public Support Using Gene Drives in Agriculture? and what Do They Want to Know? Sci. Adv. 5 (9), eaau8462. 10.1126/sciadv.aau8462 31535017 PMC6739092

[B21] Kandul N. P. Liu J. Sanchez C H. M. Marshall S. L. J. M. Akbari O. S. (2019). Transforming Insect Population Control with Precision Guided Sterile Males with Demonstration in Flies. Nat. Commun. 10 (1), 84–12. 10.1038/s41467-018-07964-7 30622266 PMC6325135

[B22] Kuzma J. Gould F. Brown Z. Collins J. Delborne J. Frow E. (2018). A Roadmap for Gene Drives: Using Institutional Analysis and Development to Frame Research Needs and Governance in a Systems Context. J. Responsible Innovation 5 (Suppl. 1), S13–S39. 10.1080/23299460.2017.1410344

[B23] Kyrou K. Hammond A. M. Galizi R. Kranjc N. Burt A. Beaghton A. K. (2018). A CRISPR-Cas9 Gene Drive Targeting Doublesex Causes Complete Population Suppression in Caged *Anopheles gambiae* Mosquitoes. Nat. Biotechnol. 36 (11), 1062–1066. 10.1038/nbt.4245 30247490 PMC6871539

[B24] Li M. Yang T. Kandul N. P. Bui M. Gamez S. Raban R. (2020). Development of a Confinable Gene Drive System in the Human Disease Vector *Aedes aegypti* . Elife 9, e51701. 10.7554/eLife.51701 31960794 PMC6974361

[B25] Li M. Yang T. Bui M. Gamez S. Wise T. Kandul N. P. (2021). Eliminating Mosquitoes with Precision Guided Sterile Males. bioRxiv. 10.1101/2021.03.05.434167

[B26] Lobe B. (2017). “Best Practices for Synchronous Online Focus Groups,” in A New Era in Focus Group Research: Challenges, Innovation and Practice. Editors Barbour R. S. Morgan D. L. (London: Palgrave Macmillan UK), 227–250. 10.1057/978-1-137-58614-8_11

[B27] Long K. C. Alphey L. Annas G. J. Bloss C. S. Campbell K. J. Champer J. (2020). Core Commitments for Field Trials of Gene Drive Organisms. Science 370 (6523), 1417–1419. 10.1126/science.abd1908 33335055

[B28] MacDonald E. A. Balanovic J. Edwards E. D. Abrahamse W. Frame B. Greenaway A. (2020a). Public Opinion towards Gene Drive as a Pest Control Approach for Biodiversity Conservation and the Association of Underlying Worldviews. Environ. Commun. 14 (7), 904–918. 10.1080/17524032.2019.1702568

[B29] MacDonald E. A. Edwards E. Balanovic J. Medvecky F. (2020b). Underlying Beliefs Linked to Public Opinion about Gene Drive and Pest-specific Toxin for Pest Control. Wildl. Res. 48 (1), 30–37. 10.1071/WR19149

[B30] MacDonald E. A. Neff M. B. Edwards E. Medvecky F. Balanovic J. (2021). Conservation Pest Control with New Technologies: Public Perceptions. J. R. Soc. New Zealand 52 (1), 95–107. 10.1080/03036758.2020.1850481 PMC11407512 39440009

[B31] Macnaghten P. (2017). “Focus Groups as Anticipatory Methodology: a Contribution from Science and Technology Studies towards Socially Resilient Governance,” in A New Era in Focus Group Research. Editors Barbour R. S. Morgan D. L. (London: Palgrave Macmillan), 343–363. 10.1057/978-1-137-58614-8_16

[B32] Marshall J. M. Touré M. B. Traore M. M. Famenini S. Taylor C. E. (2010). Perspectives of People in Mali toward Genetically-Modified Mosquitoes for Malaria Control. Malar. J. 9 (1), 128. 10.1186/1475-2875-9-128 20470410 PMC2881074

[B33] Metzger M. E. Hardstone Yoshimizu M. Padgett K. A. Hu R. Kramer V. L. (2017). Detection and Establishment of *Aedes aegypti* and *Aedes albopictus* (Diptera: Culicidae) Mosquitoes in California, 2011-2015. J. Med. Entomol. 54 (3), 533–543. 10.1093/jme/tjw237 28399270

[B34] National Academies of Sciences Engineering and Medicine (2016). Gene Drives on the Horizon: Advancing Science, Navigating Uncertainty, and Aligning Research with Public Values. Washington, D.C.: National Academies Press. 27536751

[B35] Oye K. A. Esvelt K. Appleton E. Catteruccia F. Church G. Kuiken T. (2014). Regulating Gene Drives. Science 345 (6197), 626–628. 10.1126/science.1254287 25035410

[B36] Pew Initiative on Food Biotechnology (2004). Bugs in the System? Issues in the Science and Regulation of Genetically Modified Insects. Washington, DC: Pew Charitable Trusts Available at: https://www.pewtrusts.org/en/research-and-analysis/reports/2004/01/22/bugs-in-the-system-issues-in-the-science-and-regulation-of-genetically-modified-insects .

[B37] Schairer C. E. Najera J. James A. A. Akbari O. S. Bloss C. S. (2021). Oxitec and MosquitoMate in the United States: Lessons for the Future of Gene Drive Mosquito Control. Pathog. Glob. Health. 10.1080/20477724.2021.1919378 PMC8592615 34313556

[B38] Schairer C. E. Taitingfong R. Akbari O. S. Bloss C. S. (2019). A Typology of Community and Stakeholder Engagement Based on Documented Examples in the Field of Novel Vector Control. Plos Negl. Trop. Dis. 13 (11), e0007863. 10.1371/journal.pntd.0007863 31765377 PMC6901234

[B39] Schairer C. E. Triplett C. Buchman A. Akbari O. S. Bloss C. S. (2020). Interdisciplinary Development of a Standardized Introduction to Gene Drives for Lay Audiences. BMC Med. Res. Methodol. 20, 273. Available at: https://bmcmedresmethodol.biomedcentral.com/articles/10.1186/s12874-020-01146-0#Sec19 . 10.1186/s12874-020-01146-0 33153449 PMC7643426

[B40] Yeo S. K. Brossard D. (2017). The (Changing) Nature of Scientist–media Interactions: A Cross-National Analysis. New York: Oxford University Press.

